# Evaluation of Spinning Cone Column Distillation as a Strategy for Remediation of Smoke Taint in Juice and Wine

**DOI:** 10.3390/molecules27228096

**Published:** 2022-11-21

**Authors:** Carolyn Puglisi, Renata Ristic, Jamie Saint, Kerry Wilkinson

**Affiliations:** 1 Department of Wine Science, Waite Research Institute, The University of Adelaide, PMB 1, Glen Osmond, SA 5064, Australia; 2 Australian Vintage Limited, 2 Queens Place, Balmain, NSW 2041, Australia

**Keywords:** distillation, Rate-All-That-Apply, volatile phenols, volatile phenol glycoconjugates

## Abstract

Where vineyard exposure to bushfire smoke cannot be avoided or prevented, grape and wine producers need strategies to transform smoke-affected juice and wine into saleable product. This study evaluated the potential for spinning cone column (SCC) distillation to be used for the remediation of ‘smoke taint’. Compositional analysis of ‘stripped wine’ and condensate collected during SCC treatment of two smoke-tainted red wines indicated limited, if any, removal of volatile phenols, while their non-volatile glycoconjugates were concentrated due to water and ethanol removal. Together with the removal of desirable volatile aroma compounds, this enhanced the perception of smoke-related sensory attributes; i.e., smoke taint intensified. Stripped wines also became increasingly sour and salty as ethanol (and water) were progressively removed. A preliminary juice remediation trial yielded more promising results. While clarification, heating, evaporation, deionization and fermentation processes applied to smoke-tainted white juice gave ≤3 µg/L changes in volatile phenol concentrations, SCC distillation of smoke-tainted red juice increased the volatile phenol content of condensate (in some cases by 3- to 4-fold). Deionization of the resulting condensate removed 75 µg/L of volatile phenols, but fermentation of reconstituted juice increased volatile phenol concentrations again, presumably due to yeast metabolism of glycoconjugate precursors. Research findings suggest SCC distillation alone cannot remediate smoke taint, but used in combination with adsorbents, SCC may offer a novel remediation strategy, especially for tainted juice.

## 1. Introduction

Around the world, climate change is increasingly impacting grape and wine production [1,2]. Grapegrowers and winemakers are not only being challenged by prolonged drought and heatwaves [2], but where bushfires occur in or near wine regions, by the consequences of vineyard exposure to smoke [3,4,5]. Wine made from smoke-affected grapes can exhibit unpleasant smoky, medicinal and ashy sensory characters [6,7,8,9], depending on the density of smoke [10,11], and the duration and (phenological) timing of smoke exposure [11,12,13,14]. Strategies that transform smoke-affected juice and wine into a saleable product are needed to help offset revenue losses incurred due to ‘smoke taint’, where vineyard smoke exposure cannot be avoided or prevented.

Volatile phenols (e.g., guaiacols, cresols and syringols) have been identified as compositional markers of smoke taint, in both free and glycosylated forms [6,7,8,9,10,11,12,13,14,15,16,17,18], and various strategies that mitigate either their uptake by grapes [19,20,21,22,23,24,25] or their presence in wine [4,26,27,28,29,30] have been evaluated. Currently, the most promising mitigation strategy involves enclosing grape bunches in activated carbon fabric, thereby preventing smoke contamination of grapes [24,25]. However, several shortcomings need to be addressed for this approach to be viable for use by industry in commercial vineyards [24]. As such, the use of adsorbents (e.g., activated carbon) which can be added directly to juice or wine [28,29], or used in combination with nanofiltration [30], remains the most effective strategy for amelioration of smoke taint.

**Figure 1:**
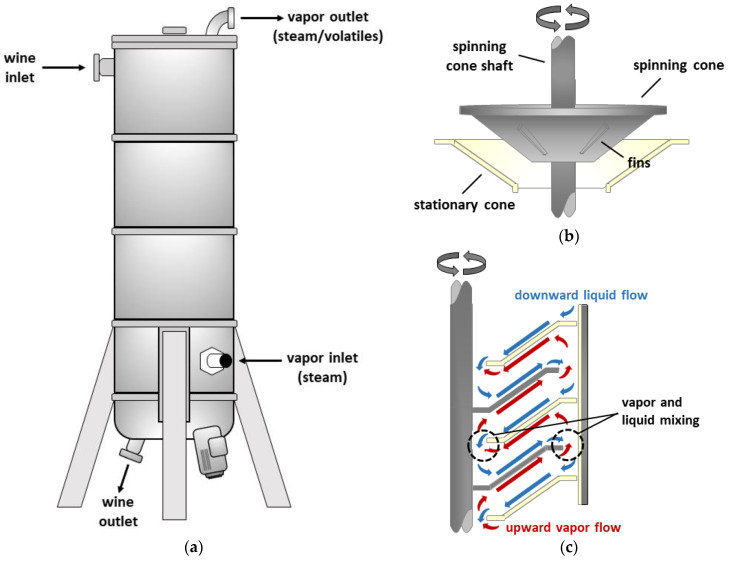
Schematic diagram of: (**a**) a spinning cone column distillation system; (**b**) a set of rotating and stationary cones; and (**c**) the downward flow of liquid and upward flow of vapor within the spinning cone column during operation.

Spinning cone column (SCC) distillation is a low temperature separation process that isolates volatile compounds from liquids, using steam, under vacuum [31,32,33,34]. The column consists of a vertical stainless steel vessel, comprising alternating stationary cones attached to the inner wall of the column, and spinning cones attached to a central rotating shaft (Figure 1). When liquid is introduced to the top of the column, it forms a thin film as it flows down the first stationary cone, and into the base of the first spinning cone, due to gravity. Centrifugal force then causes the liquid to flow across the surface of the spinning cone, i.e., upwards and outwards, again as a thin film, before it becomes airborne and falls onto the next stationary cone. This cycle repeats, resulting in the downward flow of liquid. At the same time, steam is introduced at the base of the column, creating a counter-current (upward) flow. Volatile compounds are ‘stripped’ into the vapor phase as the steam passes over the surface of the thin films and mixes with airborne liquid droplets. The addition of fins to the underside of each spinning cone (Figure 1) increases turbulence in both the liquid and vapor phases, enhancing mass transfer rates [31,34]. The volatile-enriched vapor that flows out the top of the column is passed through a condensing system and recovered in a concentrated liquid form (i.e., as ‘condensate’), while the ‘stripped’ liquid that remains is collected from the bottom of the column.

The mass transfer performance of SCC systems has been studied using various modelling techniques [34,35,36,37,38], but it is the efficient removal of volatiles at low operating temperatures (typically <30–40 °C [39])—which mitigates thermal degradation of heat labile compounds—that enables food and beverage applications of SCC distillation. These include flavour extraction/aroma recovery of fruit juice, tea and coffee [32,34,35], and alcohol adjustment of wine [33,39,40,41]. Remediation of smoke taint in juice and wine is complicated by the presence of both volatile phenols and their non-volatile glycoconjugates. In a study involving dealcoholization of wine by SCC distillation [33], the use of low temperatures resulted in preservation of flavonols, flavan-3-ols, anthocyanins, and non-flavonoids, during ethanol removal. Previous research has demonstrated the thermal stability of glycosylated aroma compounds [42]. Nevertheless, this study investigated the partitioning of free and glycosylated volatile phenols during SCC treatment of smoke-affected juice and wine, to evaluate SCC distillation as a strategy for remediation of smoke taint.

## 2. Results and Discussion

To evaluate the potential for SCC distillation to be used to remediate the sensory impact of smoke taint, two wines with elevated concentrations of free and glycosylated volatile phenols, and perceivable smoke-related sensory characteristics, were processed using an industrial scale SCC system. The composition of wine, and stripped wine and condensate fractions collected after 1%, 13–14% and 29% stripping (i.e., removal of 1%, 13–14% and 29% of the initial wine volume, as a volatile extract), were compared, along with the sensory profiles of wine and stripped wine. Since SCC distillation had to be performed on an industrial scale, it was not possible to replicate treatment of individual wines. Instead, SCC treatment was undertaken on two different smoke-tainted wines: a 2020 Shiraz Sangiovese (ShS) and a 2020 Petit Verdot Sangiovese (PVS).

### 2.1. Influence of SCC Distillation on Wine Composition

The basic composition (i.e., alcohol, residual sugar, pH, titratable acidity (TA), volatile acidity (VA), malic acid, wine color and hue, and total phenolics measurements) of wine and stripped wine are reported in Table 1. As expected, SCC distillation resulted in the progressive removal of ethanol, such that the initial alcohol concentrations of ShS and PVS wines (15.1 and 14.2% alcohol by volume (abv), respectively) were reduced to 0.3% abv in the corresponding 29% stripped wines (Table 1). Small changes in residual sugar (≤0.2 g/L) and pH (0.2) were observed, but were not considered to be meaningful from a sensory perspective. Similarly, small changes in VA, from 0.53 to 0.48 g/L for ShS and 0.36 to 0.48 g/L for PVS, were not considered to be significant. However, concentration of non-volatile organic acids, anthocyanins and tannins resulted in substantial increases in TA, malic acid (for ShS), wine color and total phenolics following 29% stripping; typically 30–40% increases were observed, (approximating the degree of concentration achieved via SCC treatment). Wine hue was not affected.

Only small changes in volatile phenol concentrations (i.e., ≤5 µg/L) were observed in stripped wines following SCC distillation (Table 2). Guaiacol concentrations initially increased (by 1–4 µg/L) with 1 and 13–14% stripping, but decreased by 3 and 5 µg/L (relative to untreated ShS and PVS wines) following 29% stripping. In contrast, syringol concentrations increased by 5 and 2 µg/L for SCC treatment of ShS and PVS wines, respectively. The concentrations of other volatile phenols changed by ≤1 µg/L following 29% stripping (Table 2), with the exception of *o*-cresol, which decreased by 2 µg/L, and *p*-cresol, which increased by 2 µg/L, following 29% stripping of ShS. Increases in volatile phenol levels reflect concentration due to the removal of ethanol and water [39], whereas decreases suggest volatile phenol removal. This was evident when changes in guaiacol and syringol concentration (and their rutinoside and gentiobioside) were compared with concentrations predicted in stripped wine as a consequence of changes in volume (i.e., accounting for concentration due to removal of 1%, 13–14% and 29% of the initial wine volume, with SCC treatment). Measured guaiacol concentrations initially followed predicted concentrations, but deviated as SCC treatment progressed (Figure S1), indicating partial removal of guaiacol. In the case of syringol, guaiacol rutinoside and syringol gentiobioside, measured and predicted concentrations were comparable (Figure S1), confirming their retention (and concentration) in stripped wine. The relative removal and/or concentration of volatile phenols (in free and glycosylated forms) during SCC treatment is also apparent when the composition of condensate fractions are considered (Table 2). 

Guaiacol was detected in all ShS and PVS condensate fractions, with concentrations increasing as a function of the degree of stripping; i.e., from 30 and 35 µg/L after 1% stripping to 80 and 65 µg/L after 29% stripping (of ShS and PVS, respectively). Significantly lower levels of 4-methylguaiacol and *o*-, *m*- and *p*-cresols were present in condensates, i.e., 5–10 µg/L, predominantly in condensate collected after 13–14% and/or 29% stripping, whereas syringol and 4-methylsyringol were not detected in any condensate fractions. The volatile phenol composition of condensate fractions can be explained by differences in volatility (Table S1). Of the volatile phenols measured, syringol and 4-methylsyringol have the highest boiling points (260 and 268 °C) and lowest vapor pressures (0.006 and 0.005 mm Hg at 25 °C). As such, they are not extracted during SCC distillation, even at higher stripping rates. In contrast, guaiacol has the highest vapor pressure (0.179 mm Hg at 25 °C), and is therefore extracted from wine to a greater extent than 4-methylguaiacol and the cresols. The SCC distillation conditions employed in the current study (i.e., vacuum pressure, feed and steam flow rates, operating temperatures and strip rates) were those routinely used to achieve dealcoholization of wine. These parameters could, however, be manipulated to facilitate isolation of volatile phenols, albeit, this might require stripping beyond 29% and the use of higher operating temperatures.

Glycosylation of volatile phenols renders them non-volatile. As such, SCC treatment was not expected to result in the removal of volatile phenol glycoconjugates. The concentrations of the volatile phenol glycoconjugates that were measured (being the rutinosides of guaiacol, 4-methylguaiacol, phenol and cresol, and gentiobiosides syringol and 4-methylsyringol) increased with SCC distillation (Table 1), as predicted; no glycoconjugates were detected in condensate fractions. Again, changes in volatile phenol glycoconjugates seemingly followed the degree of stripping, and thus, reflected a concentration effect. Compositional analyses provided no evidence of any appreciable hydrolysis of volatile phenol glycoconjugates under the (relatively mild) operating conditions employed during SCC treatment, in agreement with previous research that demonstrated the relative stability of volatile phenol glycoconjugates [42].

These results suggest SCC distillation alone does not present a viable strategy for remediation of smoke tainted wine, since neither volatile phenols nor their glycoconjugates were removed to an extent that would enhance wine aroma, flavor and/or quality. However, it could potentially be used in combination with other remediation strategies, e.g., the addition of adsorbents, such as activated carbon, to facilitate treatment of wine fractions rather than whole wine, thereby mitigating negative impacts of these treatments on desirable wine constituents (e.g., loss of varietal characters).

### 2.2. Influence of SCC Distillation on Wine Sensory Profiles

Wines made from smoke-affected grapes often exhibit diminished fruit characters due to masking by smoky, ashy aromas and flavors [6,7,8,9,10,11,24]. However, despite having elevated concentrations of smoke taint markers compounds (Table 1), the wines sourced for this study retained fruit expression, with intensity ratings for smoke-related sensory attributes being considerably lower, i.e., 0.9–2.5/7, than ratings for fruit aroma and flavor, i.e., 3.6–4.0/7 (Table S2).

**Figure 2:**
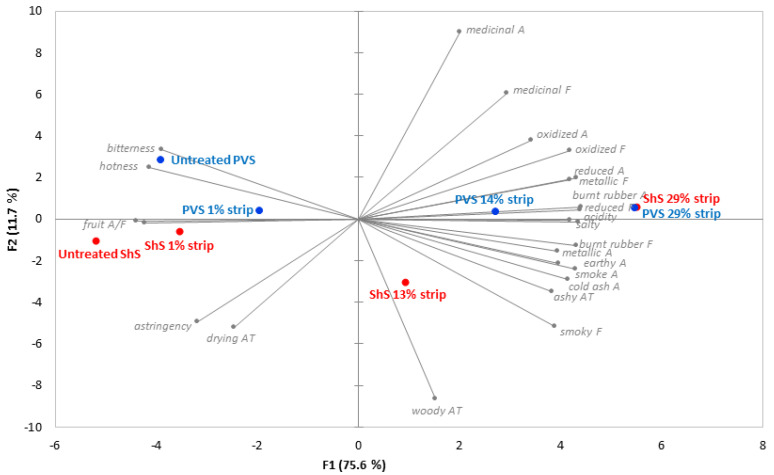
Principal component analysis biplot of mean sensory attribute ratings for smoke-tainted Shiraz Sangiovese (ShS) and Petit Verdot Sangiovese (PVS) wines, before and after spinning cone column distillation. A = aroma, F = flavor, AT = aftertaste.

Significant differences were perceived in the sensory profiles of ShS and PVS wines before and after SCC distillation (Figure 2, Table S2). The intensity of fruit aroma and flavor diminished, while the perception of smoke, cold ash and burnt rubber aromas, and smoky, medicinal and burnt rubber flavors intensified as a function of stripping. The characteristic ashy aftertaste often associated with smoke-tainted wines also became more apparent with SCC treatment. The loss of fruit expression can be attributed to the removal of desirable aroma volatiles, which occurs as an outcome of SCC distillation, in addition to dealcoholization [39]. Gas chromatography-mass spectrometry (GC-MS) analysis confirmed the presence of fermentation volatiles (alcohols and esters) in each of the condensate fractions (Table S3); their lower boiling points (relative to volatile phenols) likely aided their removal during SCC distillation (Tables S1 and S2). The extraction of volatiles responsible for fruit aroma and flavor, together with concentration of free and glycosylated volatile phenols provides a plausible explanation for the perceived intensification of smoke taint following SCC treatment. The biplot generated from principal component analysis of sensory data (Figure 2) shows clear separation of wines along the x-axis according to the degree of stripping; with the first principal component accounting for 75.6% of the total variance. Untreated wines were positioned furthest to the left, reflecting predominant fruit expression, while stripped wines moved progressively to the right, due to the intensification of smoke taint sensory characters.

Based on a preliminary bench-top tasting trial, reduced aroma and flavor, oxidized aroma and flavor, and saltiness were included as attributes for evaluation during sensory analysis (Tables S2 and S4). Intensity ratings for reduced and oxidized characters were initially low at 0.78–1.70/7.0 for untreated wines, however, small but statistically significant increases in ratings were obtained following SCC distillation (Table S2); the exception being ratings for oxidized aroma in PVS samples, which were not significantly different (Table S2). Despite ‘reductive’ and ‘oxidative’ being contrasting terms, the sensory panel perceived differences in the intensities of the descriptors represented by these terms. Nevertheless, it is unclear to what extent these changes truly reflect either reduction or oxidation arising from SCC distillation vs. the panel’s perception of underlying characters being enhanced by the loss of fruit expression (due to the aforementioned removal of desirable aroma volatiles).

The impact of SCC distillation on taste and mouthfeel attributes was more obvious (Figure 2, Table S2). The perception of acidity (sourness) and saltiness was clearly enhanced; not only due to the concentration of non-volatile organic acids and salts, but also the loss of ethanol, which has been shown to moderate acid perception [43,44]. The removal of ethanol also accounts for the diminished perception of hotness. Small but significant decreases in bitterness, and drying and astringent characters were perceived following SCC distillation of the PVS wine, despite the likely concentration of (non-volatile) phenolic compounds [33], and observed increased in total phenolics (Table 1).

Dealcoholization of wine by SCC distillation typically occurs in two steps; volatile aroma compounds are removed first, and then ethanol, with the aroma fraction being captured (as condensate) for blending with the de-alcoholized product to restore aroma and flavor [33,39,41]. Grape juice concentrate can also be used to enhance body (viscosity) and/or fruit expression. In the current study, no such manipulations were made to stripped wine fractions prior to sensory analysis, but this could be considered, in combination with the aforementioned use of adsorbents, to help mitigate the sensory impacts of smoke on wine aroma, flavor and quality.

### 2.3. Influence of SCC Distillation on Juice Composition

**Figure 3:**
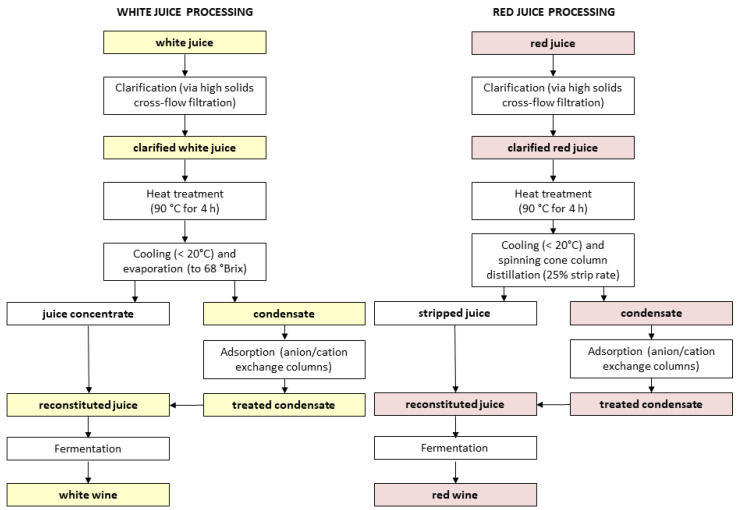
Flowcharts of processes used to remediate white and red juices derived from smoke-affected grapes. Colored boxes represent samples collected for compositional analysis.

In a parallel, but preliminary trial, remediation of smoke-tainted juice was evaluated by comparing compositional changes in samples collected at different stages of processing (Figure 3). White juice processing involved evaporation, whereas red juice underwent SCC distillation (with 25% stripping). In each case, the resulting condensates were subsequently subjected to an ion exchange column treatment (to remove volatile phenols via solid-phase adsorption), before being blended with their juice concentrate. Reconstituted juices were then fermented to produce wine.

Free and glycosylated volatile phenols were detected in both juices (Table 3), but higher concentrations were observed for red juice, which likely reflects the relative abundance of naturally occurring (‘baseline’) volatile phenols in red vs. white cultivars [45], as well as the duration of skin contact [26] and/or smoke exposure [11,12]. Clarification had little effect on juice composition, with free and glycosylated volatile phenol concentrations changing by ≤1 and 5 µg/L, respectively. However, while evaporation of white juice yielded similar volatile phenol concentrations (only syringol increased, from <1 to 2 µg/L), volatile phenol glycoconjugates were not detected in white juice condensate (again, because they are non-volatile). Following ion exchange column treatment (which was performed to remove volatile phenols via solid-phase adsorption), only 2 µg/L of guaiacol remained in the condensate; no other volatile phenols were detected, suggesting they were indeed removed by the ion exchange column. After blending, syringol was detected in the reconstituted juice (at 3 µg/L), which also comprised volatile phenol glycoconjugates at levels comparable to those observed in the original juice; only syringol gentiobioside had decreased substantially (from 34 to 22 µg/L). Fermentation of the reconstituted white juice resulted in the release of 1–3 µg/L of guaiacol, 4-methylguaiacol and *o*-, *m*- and *p*-cresol, but neither syringol nor 4-methylsyringol were detected in the white wine. Presumably this represents partial metabolism of the glycoconjugate pool by yeast, as reported in previous studies [8]. However, wine glycoconjugate data is not available for verification. 

SCC distillation of red juice generated condensate with ~3- to 4-fold higher concentrations of guaiacol, 4-methylguaiacol, and *o*- and *m*-cresol (Table 3), while *p*-cresol and syringol, which were not detected in red juice, were present in condensate at 2 and 5 µg/L, respectively. With the exception of syringol, these results can be attributed to the direct extraction of volatile phenols from juice, given concentration changes approximate the 25% stripping rate that was applied; even the presence of 2 µg/L of *p*-cresol in condensate is reasonable if the initial juice concentration was ~0.5 µg/L (noting juice *p*-cresol was reported as ‘not detected’ (Table 3), due to the 1 µg/L limit of detection). The relative ease with which these volatile phenols were removed from juice via SCC distillation, compared with SCC treatment of wine (Table 2), likely reflects differences in vapor partitioning due to the presence of sugars [46,47], and absence of ethanol. Certainly, the ‘salting-out’ effect of sugars on the liquid-vapor partitioning of different volatile compounds has previously been reported in coffee [48], soft drink [49] and mango juice [50].

The presence of syringol in condensate (at 5 µg/L) presumably also reflects extraction from juice, however, compositional analysis of reconstituted red juice (Table 3) indicates partial hydrolysis of syringol gentiobioside occurred during heat treatment and/or SCC distillation, explaining the source of syringol, which was not detected in red juice. Nevertheless, the observed difference in syringol gentiobioside concentrations for clarified red juice and reconstituted red juice, being 22 µg/L, only accounts for evolution of 7 µg/L of syringol (assuming quantitative release due to hydrolysis). This suggests hydrolysis of other syringol glycoconjugates or, as noted in previous studies in which mass balance could not be achieved [10,17], hydrolysis of other conjugates that are yet to be identified.

SCC distillation did not result in removal of (non-volatile) glycoconjugates from the red juice, in agreement with results from SCC distillation of wine (Table 2); as such, the glycosylated volatile phenols measured in red juice were not detected in the resulting condensate (Table 3). Ion exchange column treatment of condensate achieved almost complete removal of volatile phenols; only guaiacol remained at a detectable concentration, being 1 µg/L. When the treated condensate was blended with the stripped juice, the reconstituted juice was found to have only 1–2 µg/L of guaiacol, *m*-cresol and 4-methylsyringol, and 22 µg/L of syringol (Table 3). Again, hydrolysis of syringol gentiobioside (and/or other syringol glycoconjugates/conjugates) provides a plausible explanation for the increase in syringol following SCC distillation, and is consistent with the observed decrease in syringol gentiobioside, from 85 to 62 µg/L, relative to the original red juice (Table 3). Fermentation of the reconstituted red juice also resulted in the release of up to 8 µg/L of selected volatile phenols (i.e., guaiacol, *m*- and *p*–cresol, syringol and 4-methylsyringol). Again this likely reflects metabolism of glycosylated volatile phenols during winemaking; the release of two-fold higher quantities of syringol suggests its gentiobioside (and/or other syringol glycoconjugates) might be more susceptible to hydrolysis than the glycoconjugates of other volatile phenols. However, as with the white wine, red wine glycoconjugate data was not available for reference.

Results from the juice remediation trial are promising, in that some removal of volatile phenols was achieved, but the preliminary nature of findings and need for further research (e.g., to establish whether or not treatments result in a perceivable sensory outcome), are acknowledged. Samples were collected during commercial processing and compositional analyses performed retrospectively. Wine sensory analysis was no longer possible because by then, wines had been used as blending components in commercial production. This is a limitation of the current study. Nevertheless, research findings justify further investigation into the combined use of SCC distillation and adsorbent materials capable of binding volatile phenols, for remediation of smoke taint, particularly as a strategy for remediation of smoke-tainted juice.

## 3. Materials and Methods

### 3.1. Remediation of Smoke-Tainted Wine

Two wines, a 2020 Shiraz Sangiovese and a 2020 Petit Verdot Sangiovese, were sourced from a commercial winery (Cassegrain Wines, Port Macquarie, NSW, Australia) that deemed the wines to be smoke-tainted and in need of remediation. The wines were made from Shiraz and Petit Verdot grapes harvested from vineyards in Mudgee (32°36′ S 149°34′ E) and Sangiovese grapes harvested from a vineyard in Hilltops (34°24′ S 148°25′ E), that were exposed to smoke from bushfires that burned in New South Wales during the 2019/2020 growing season (albeit, the exact duration of vineyard smoke exposure and density of smoke are not known).

Wines (~8000 L) were treated by Australian Vintage Limited (at their Buronga Hill winery/Austflavor facility; Buronga, NSW, Australia) using an industrial scale spinning cone column distillation system (SCC10000, Flavourtech, Griffith, NSW, Australia). Briefly, wines were fed into the top of the column (comprising a cone stack ~0.88 m wide and ~3.64 m high) under vacuum, with stripping steam fed into the base of the column (Figure 1). The vapor that flowed from the top of the column (steam mixed with volatile compounds stripped from the wine), was passed through a condensing system and collected as ‘condensate’, while the resulting ‘stripped wine’ was collected from the bottom of the column. Each SCC distillation treatment was conducted under operating conditions (Table 4) that achieved three distinct fractionation end points, ~1%, ~13–14% and ~29% stripping, during which condensate and stripped wine samples were collected. Condensate (~300 mL) was frozen (at −4 °C) for chemical analysis, while untreated and stripped wines (~20 L) were bottled (in 750 mL glass wine bottles, under screw-cap closures) and cellared (at 15 °C) for chemical and sensory analyses (which were performed within 3 months of SCC distillation treatment). Since SCC distillation had to be performed at industrial scale, it was not possible to replicate treatments on individual wines. Instead, SCC treatment of two wines was undertaken.

### 3.2. Remediation of Smoke-Tainted Juice

Two juices, a white juice derived from smoke-affected Sauvignon Blanc grapes and a red juice derived from smoke-affected Pinot Grigio, Tempranillo and Pinot Noir grapes, were remediated by Australian Vintage Limited (again at their Buronga Hill winery/Austflavor facility). Grapes were harvested from vineyards located in Charleston (34°55′ S 138°54′ E) in the Adelaide Hills wine region, that were known to have been exposed to smoke from the Cudlee Creek bushfire, which started on 20 December 2019 and burned until early January 2020 (as before, the exact duration of vineyard smoke exposure and density of smoke are not known). Grapes were processed according to commercial protocols and juice remediation undertaken as shown in Figure 3.

Both juices were initially clarified via high solids cross-flow filtration (Omnia Crossflow, Della Toffola Pacific, Preston, VIC, Australia) before heating at 90 °C (for 4 h). On cooling (to <20 °C), the white juice was concentrated to ~68° Brix with a CT12 Centritherm^®^ evaporator (Flavourtech, Griffith, NSW, Australia) and the resulting condensate treated via an inline anion/cation exchange process (comprising sequential food-grade ion exchange columns (2400 × 920 mm and 2800 × 1200 mm) charged with acid and base, respectively) to remove volatile phenols, before being blended with juice concentrate. The reconstituted juice was subsequently fermented to produce white wine. On cooling (to <20 °C), the red juice was subjected to spinning cone column distillation (as described above, but to a fractionation end point that achieved 25% stripping) and the resulting condensate treated via the inline anion/cation exchange process (as for the white juice condensate), before being blended with the stripped red juice; the reconstituted juice was subsequently fermented to produce red wine. During juice treatments (Figure 3), clarified juice, condensate, treated condensate, reconstituted juice, and wine samples (375 mL) were collected for chemical analysis.

### 3.3. Chemical Analysis of Juice, Condensate and Wine Samples

Alcohol, pH, titratable acidity (TA, as g/L of tartaric acid) and volatile acidity (VA, as g/L of acetic acid) were measured using a Foss WineScan analyzer (Mulgrave, VIC, Australia), while red wine color and total phenolics were measured using the modified Somers method and methyl cellulose precipitable tannin assay [51], respectively. These measurements were completed by the Australian Wine Research Institute’s (AWRI) Commercial Services Laboratory (Adelaide, SA, Australia). Residual sugar and malic acid were measured enzymatically (Boehringer-Mannheim, R-BioPharm, Darmstadt, Germany) with a liquid handling robot (CAS-3800, Corbett Robotics, Eight Mile Plain, Qld, Australia) and spectrophotometric plate reader (Infinite 200 Pro, Tecan, Männedorf, Switzerland). The AWRI’s Commercial Services laboratory also quantified volatile phenols and their glycoconjugates (in juice, condensate and wine) by GC-MS and HPLC-MS/MS, respectively; in each case using previously published stable isotope dilution assay methods [52,53]. The preparation of internal standards, method validation and instrument operating conditions are detailed in these publications, but briefly: volatile phenols were quantified with an Agilent 6890 gas chromatograph coupled to a 5973 mass selective detector (Agilent Technologies, Forest Hill, Vic., Australia), using *d*_3_-guaiacol, *d*_3_-4-methylguaiacol, *d*_7_-*o*-cresol, and *d*_3_-syringol as internal standards (limits of detection were 1–2 µg/L); and volatile phenol glycoconjugates were quantified with an Agilent 1200 high performance liquid chromatograph equipped with a 1290 binary pump coupled to an AB SCIEX Triple Quad^TM^ 4500 tandem mass spectrometer, with a Turbo V^TM^ ion source (Framingham, MA, USA), using *d*_3_-syringol gentiobioside (Toronto Research Chemicals, Toronto, ON, Canada) as an internal standard (the limit of detection was 1 µg/L).

Qualitative analysis of condensate samples collected during SCC treatment of smoke-tainted wines was performed by Metabolomics Australia (AWRI, Adelaide, SA, Australia) using headspace-solid phase microextraction (HS-SPME) [54]. Briefly, samples (diluted to ~1% abv, with water, and saturated with 2.0 g of sodium chloride) were extracted with a DVB/CAR/PDMS SPME fiber (Sigma Aldrich, North Ryde, NSW, Australia) for 15 min at 40 °C, prior to desorption (in splitless mode), at an injector temperature of 260 °C, onto an Agilent 7890 A gas chromatograph fitted with an Agilent DB-624UI column (30 m × 0.25 mm × 1.4 µm) equipped with a Gerstel MPS2 multi-purpose autosampler, and coupled to a 5975C VL mass selective detector. Helium (ultra high purity) was used as the carrier gas in constant flow mode. GC-MS data from Agilent ChemStation software (v E.02.02) were exported for processing using R statistical programming software (v4.1.1, RStudio Inc., Boston, MA, USA). Volatile compounds were tentatively identified by comparing mass spectral data with the NIST mass spectral database.

### 3.4. Sensory Analysis of Wine

The sensory profiles of untreated and stripped wines were determined using the Rate-All-That-Apply (RATA) method [55], with a panel of 50 participants (12 male and 38 female, aged between 22 and 67 years), comprising staff and students from the University of Adelaide and the AWRI, together with regular wine consumers recruited from an in-house database. Wines were initially assessed by four sensory experts to ensure the sensory attributes to be rated (i.e., aroma, flavor, taste and mouthfeel descriptors evaluated in an earlier smoke taint study [8,10]), were suitable. This resulted in the inclusion of several additional descriptors: reduced aroma and flavor; oxidized aroma and flavor; and saltiness. Prior to wine evaluation, panelists were familiarized with both the RATA process and the list of sensory attributes (Table S4). Evaluations were then conducted in sensory booths at 22–23 °C under sodium lights, with wine aliquots (30 mL) presented monadically, in a randomized order, in covered, 4-digit coded 215 mL stemmed International Organization for Standardization wine glasses. Panelists rated the intensity of each sensory attribute using seven-point Likert scales (where 0 = not detected, 1 = ‘extremely low’ and 7 = ‘extremely high’). Panelists rinsed thoroughly with pectin solution (1 g/L) and rested for at least 1 min between samples to mitigate sensory fatigue; water and plain crackers were also provided as palate cleansers. Data was acquired with Red Jade software (Redwood Shores, CA, USA).

### 3.5. Data Analysis

Sensory data were analyzed by two-way ANOVA using participants as a random factor and wines as a fixed factor, with Fischer’s LSD post hoc test (*p* ≤ 0.05), to determine significant differences between wines, using XLSTAT (version 2018.1.1, Addinsoft, New York, USA). Mean comparisons were performed by least significant difference (LSD) multiple comparison test at *p* < 0.05. Principal component analysis (PCA) of sensory data was also performed using XLSTAT.

## Figures and Tables

**Table 1:** Basic chemistry of smoke-tainted wines, before and after spinning cone column distillation treatment.

	Shiraz Sangiovese				Petit Verdot Sangiovese			
	Untreated	1% Strip	13% Strip	29% Strip	Untreated	1% Strip	14% Strip	29% Strip
alcohol (% abv)	15.1	14.5	7.9	0.3	14.2	13.8	6.8	0.3
residual sugar (g/L)	1.4	1.4	1.6	1.4	0.7	0.7	0.8	0.8
pH	3.7	3.7	3.6	3.5	3.7	3.7	3.6	3.5
TA (g/L)	6.3	6.4	7.2	8.5	5.7	5.7	6.4	7.3
VA (g/L)	0.53	0.50	0.42	0.48	0.36	0.36	0.38	0.48
malic acid (g/L)	1.35	1.42	1.67	1.99	<0.10	<0.10	<0.10	<0.10
wine color (au)	6.5	6.6	7.7	9.2	4.2	4.4	5.1	5.9
wine hue	0.87	0.89	0.89	0.87	0.96	0.96	0.91	0.95
phenolics (au)	48	49	55	65	32	33	38	43

**Table 2:** Concentrations of volatile phenols and their glycoconjugates (µg/L) in smoke-tainted Shiraz Sangiovese and Petit Verdot Sangiovese wines, before and after spinning cone column distillation, and in their corresponding condensates.

	Treatment	Guaiacol	4-Methyl Guaiacol	*o*-Cresol	*m*-Cresol	*p*-Cresol	Syringol	4-Methyl Syringol	GuR	4MGR	PhR	CrR	SyrGB	4MSGB
Shiraz Sangiovese	Untreated	49	7	8	10	11	13	3	41	37	26	27	112	7
	1% strip	50	6	7	9	12	13	3	40	34	24	26	107	7
	13% strip	52	7	7	10	13	15	3	47	42	28	32	127	8
	29% strip	46	6	6	10	13	18	4	55	51	35	38	152	11
	1% strip condensate	30	nd	nd	nd	nd	nd	nd	nd	nd	nd	nd	nd	nd
	13% strip condensate	35	nd	5	nd	nd	nd	nd	nd	nd	nd	nd	nd	nd
	29% strip condensate	80	10	10	5	10	nd	nd	nd	nd	nd	nd	nd	nd
Petit Verdot Sangiovese	Untreated	55	10	7	11	12	15	3	39	33	20	26	94	6
	1% strip	56	10	7	11	12	14	3	42	35	23	25	99	6
	14% strip	59	11	7	12	13	16	3	50	43	26	32	114	7
	29% strip	50	9	6	11	13	17	3	61	51	32	38	138	8
	1% strip condensate	35	5	nd	nd	nd	nd	nd	nd	nd	nd	nd	nd	nd
	14% strip condensate	45	5	5	nd	5	nd	nd	nd	nd	nd	nd	nd	nd
	29% strip condensate	65	10	10	5	5	nd	nd	nd	nd	nd	nd	nd	nd

Glycosides measured as syringol glucose-glucoside (gentiobioside) equivalents; nd = not detected. GuR = guaiacol rutinoside; 4MGR = 4-methylguaiacol rutinoside; PhR = phenol rutinoside; CrR = cresol rutinoside; SyrGB = syringol glucose-glucoside; 4MSGB = 4-methylsyringol glucose-glucoside.

**Table 3:** Concentrations of volatile phenols and their glycoconjugates (µg/L) in samples collected during remediation of smoke-tainted white and red juice.

Treatment	Guaiacol	4-Methyl Guaiacol	*o*-Cresol	*m*-Cresol	*p*-Cresol	Syringol	4-Methyl Syringol	GuR	4MGR	PhR	CrR	SyrGB	4MSGB
white juice	4	nd	1	2	1	nd	nd	27	23	11	28	34	4
clarified white juice	4	1	2	2	nd	nd	nd	25	22	12	25	29	4
condensate (pre-IEX)	4	1	1	2	nd	2	nd	nd	nd	nd	nd	nd	nd
condensate (post-IEX)	2	nd	nd	nd	nd	nd	nd	nd	nd	nd	nd	nd	nd
reconstituted white juice	2	nd	nd	nd	nd	3	nd	27	23	11	28	22	3
white wine	5	1	2	2	2	nd	nd	na	na	na	na	na	na
red juice	10	2	3	3	nd	nd	nd	19	27	10	23	85	11
clarified red juice	9	2	3	3	nd	nd	nd	19	27	10	24	84	11
condensate (pre-IEX)	42	6	12	9	2	5	nd	nd	nd	nd	nd	nd	nd
condensate (post-IEX)	1	nd	nd	nd	nd	nd	nd	nd	nd	nd	nd	nd	nd
reconstituted red juice	2	nd	nd	1	nd	22	2	20	28	11	24	62	10
red wine	4	nd	nd	2	1	30	3	na	na	na	na	na	na

Glycosides measured as syringol glucose-glucoside (gentiobioside) equivalents; nd = not detected; na = not available. IEX = ion exchange; GuR = guaiacol rutinoside; 4MGR = 4-methylguaiacol rutinoside; PhR = phenol rutinoside; CrR = cresol rutinoside; SyrGB = syringol glucose-glucoside; 4MSGB = 4-methylsyringol glucose-glucoside.

**Table 4:** System operating conditions during industrial scale spinning cone column distillation treatment of smoke-tainted Shiraz Sangiovese and Petit Verdot Sangiovese wines.

Operating	Shiraz Sangiovese			Petit Verdot Sangiovese		
	1% Strip	13% Strip	29% Strip	1% Strip	14% Strip	29% Strip
feed flow (L/h)	2978	1823	1793	3173	1879	1852
inlet temperature (°C)	15.9	16.5	16.7	15.6	16.0	16.3
top vapour temperature (°C)	28.5	31.1	37.1	28.5	31.8	37.3
bottom product temperature (°C)	31.4	36.2	48.4	31.5	37.2	48.9
vacuum pressure (kPa)	94.9	95.0	95.0	95.0	94.9	95.0
drive motor current (A)	11.5	11.0	12.8	11.0	10.4	13.4
steam flow rate (kg/h)	17	127	412	20	145	432
condensate strip rate (%)	1.1	13.0	29.0	1.0	14.3	29.0
condensate flow rate (L/h)	32	233	519	32	256	522
condensor temperature (°C)	3	2	4	3	2	5

## Data Availability

All data are included in the article and/or Supplementary Materials.

## Supplementary Materials

The following supporting information can be downloaded at: https://www.mdpi.com/article/10.3390/molecules27228096/s1, Table S1: Chemical structures and physical properties of smoke-derived volatile phenols.; Table S2: Mean intensity ratings for sensory attributes of smoke-tainted Shiraz Sangiovese and Petit Verdot Sangiovese wines, before and after spinning cone column distillation.; Table S3: Concentrations of fermentation volatiles (µg/L) detected in condensate derived from spinning cone column distillation of smoke-tainted Shiraz Sangiovese and Petit Verdot Sangiovese wines [56,57]; Table S4: Aroma and palate attributes evaluated during sensory analysis; Figure S1: Predicted and actual concentrations of (**a**,**b**) guaiacol (Gu) and syringol (Syr), and (**c**,**d**) their glycoconjugates (GuR and SyrGB), in smoke-tainted (**a**,**c**) Shiraz Sangiovese and (**c**,**d**) Petit Verdot Sangiovese wines, before and after SCC distillation.

## Sample Availability

Samples of compounds are not available from the authors.
