# Supplementary material for: Evaluation of Spinning Cone Column Distillation as a Strategy for Remediation of Smoke Taint in Juice and Wine
Source: Molecules. 2022 Nov 21;27(22):8096. doi: 10.3390/molecules27228096 (PMC9697475; doi:10.3390/molecules27228096)
Supplement: Supplementary file 1 [file molecules-27-08096-s001.zip › molecules-1959740-supplementary.pdf]

Evaluation of Spinning Cone Column Distillation as a Strategy for Remediation of Smoke Taint in Juice and Wine

Carolyn Puglisi [1], Renata Ristic [1], Jamie Saint [2] and Kerry Wilkinson [1,*]

[1] Department of Wine Science, Waite Research Institute, The University of Adelaide, PMB 1, Glen Osmond, SA 5064, Australia
[2] Australian Vintage Limited, 2 Queens Place, Balmain, NSW 2041, Australia
* Correspondence: kerry.wilkinson@adelaide.edu.au (K.W.); Tel: +61-8-8313-7360

Table S1. Chemical structures and physical properties of smoke-derived volatile phenols.

	**Guaiacol**	**4-Methyl Guaiacol**	***o*-Cresol**	***m*-Cresol**	***p*-Cresol**	**Syringol**	**4-Methyl Syringol**
Structure	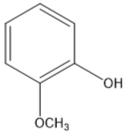	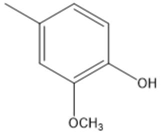	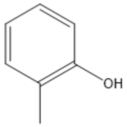	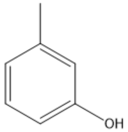	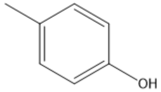	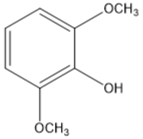	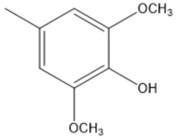
Molecular weight (amu)	124.14	186.25	108.14	108.14	108.14	154.17	168.19
Boiling point (°C)	205	221	191	202	202	260	268
Vapor pressure (mm Hg at 25°C)	0.179	0.078	0.030[1]	0.110	0.110	0.006	0.005

Values sourced from The Good Scents Company (www.thegoodscentscompany.com, accessed on 28th August 2022), except for the vapor pressure for 4-methylsyringol, which was sourced from ChemBK (www.chembk.com/en, accessed on 28th August 2022). [1] Vapor pressure (mm Hg at 20 °C).

Table S2. Mean intensity ratings for sensory attributes of smoke-tainted Shiraz Sangiovese and Petit Verdot Sangiovese wines, before and after spinning cone column distillation.

	Shiraz Sangiovese				P	Petit Verdot Sangiovese				P
	Control	1% strip	13% strip	29% strip		Control	1% strip	14% strip	29% strip	
fruit aroma	4.04 a	3.64 ab	3.38 b	2.82 c	<0.0001	3.94 a	3.30 b	3.12 b	2.64 c	<0.0001
smoke aroma	2.20 b	2.64 b	3.42 a	3.88 a	<0.0001	2.14 c	2.78 bc	3.32 ab	3.78 a	<0.0001
cold ash aroma	1.94 c	2.48 bc	3.12 ab	3.34 a	<0.0001	1.88 b	2.42 b	3.32 a	3.30 a	<0.0001
earthy aroma	2.48	2.24	2.64	3.04	ns	2.06 c	2.50 bc	2.64 ab	3.12 a	0.002
medicinal aroma	1.80	1.88	1.80	2.48	ns	2.44	2.20	2.00	2.38	ns
burnt rubber aroma	0.88 c	1.20 c	2.00 b	2.68 a	<0.0001	1.28 b	1.66 b	2.36 a	2.76 a	<0.0001
reduced aroma	0.78 c	1.22 bc	1.50 b	2.64 a	<0.0001	1.20 b	1.30 b	2.02 a	2.52 a	<0.0001
oxidized aroma	1.14 b	1.22 b	1.54 b	2.52 a	<0.0001	1.70	1.34	1.52	1.88	ns
fruit flavor	3.78 a	3.54 ab	2.92 cd	2.24 e	<0.0001	3.62 a	3.26 b	2.76 c	2.04 d	<0.0001
smoky flavor	2.52 cd	2.58 cd	3.30 ab	3.30 ab	0.012	2.06 d	2.76 bc	3.08 abc	3.36 a	<0.0001
medicinal flavor	1.86 b	1.82 b	2.24 ab	2.76 a	0.017	2.54 a	2.46 ab	2.58 a	2.44 abc	ns
burnt rubber flavor	1.08 b	1.26 b	1.82 a	2.10 a	0.001	1.24 c	1.34 bc	1.80 ab	1.92 a	0.015
reduced flavor	0.84 c	1.10 bc	1.40 ab	1.88 a	0.0001	1.02 c	1.06 bc	1.60 ab	1.94 a	0.003
oxidized flavor	1.20 b	1.18 b	1.64 b	2.96 a	<0.0001	1.64 c	1.66 c	2.30 b	2.90 a	<0.0001
ashy aftertaste	2.48 cd	2.92 bc	3.16 b	3.26 ab	<0.0001	2.08 c	2.78 b	3.26 ab	3.80 a	<0.0001
woody aftertaste	2.58	2.48	3.02	2.68	ns	2.44	2.60	2.62	2.56	ns
metallic	1.52 b	1.54 b	1.94 ab	2.30 a	0.015	1.80	1.68	2.16	2.16	ns
acidity	3.96 c	3.68 c	4.48 b	5.90 a	<0.0001	3.62 c	3.70 c	4.84 b	5.78 a	<0.0001
bitterness	3.44	3.54	3.08	3.10	ns	3.60 a	3.48 ab	2.96 c	3.02 bc	0.014
saltiness	2.04 c	2.22 c	2.96 b	3.80 a	<0.0001	2.14 c	2.48 c	2.98 b	3.90 a	<0.0001
hotness	3.78 a	3.52 a	2.44 b	2.42 b	<0.0001	3.62 a	3.58 a	2.40 b	2.38 b	<0.0001
drying	3.90	3.68	3.82	3.68	ns	3.54 ab	3.62 a	3.12 b	3.16 b	0.018
astringency	3.88	3.70	3.74	3.40	ns	3.54 a	3.62 a	3.02 b	3.26 ab	0.020

Values are means of ratings from 50 panelists. Different letters within rows indicate statistical significance ($P \leq 0.05$, two-way ANOVA); ns = not significant.

Table S3. Median normalized peak heights of fermentation volatiles (μg/L) detected in condensate derived from spinning cone column distillation of smoke-tainted Shiraz Sangiovese and Petit Verdot Sangiovese wines.

Volatile	Descriptors[1]	Boiling Point (°C)[2]	Shiraz Sangiovese			Petit Verdot Sangiovese			Match Factor
			1% strip	14% strip	28% strip	1% strip	14% strip	28% strip	
ethyl acetate	nail polish	77	6005[3]	9841	8623	7793[3]	7734	6764	90%
ethyl propanoate	fruity	99	119	97	86	169	101	84	91%
ethyl butanoate	fruity, strawberry	121	389	327	246	391	262	197	93%
ethyl hexanoate	green apple, fruity	168	6005[3]	7293	5681	7793[3]	6644	4925	99%
ethyl octanoate	melon, soap	208	6005[3]	7178	6574	7793[3]	7777	5735	99%
ethyl decanoate	floral, soap	245	4301	1618	1515	7104	1646	1074	99%
2-methylpropyl acetate	banana	118	28	67	84	29	57	75	59%
2-methylbutyl acetate	banana	140	662	484	320	833	516	365	83%
3-methylbutyl acetate	banana	142	3108	3072	2115	4293	3554	2709	90%
hexyl acetate	lolly	172	883	293	175	1317	335	180	90%
1-propanol	alcohol, pungent	97	36	103	114	50	99	116	86%
2-methylpropanol	wine, solvent, bitter	108	274	607	570	325	513	461	86%
2-methylbutanol	solvent	129	806	2321	2207	1325	2571	2593	90%
3-methylbutanol	harsh, nail polish, fusel	131	1591	4453	4597	2423	4396	5034	90%
hexanol	green, grass	157	211	497	353	201	353	250	90%

[1] Descriptors sourced from Wang et al. 2018 [56], Smyth 2005 [57], and publications cited therein. [2] Values sourced from The Good Scents Company (www.thegoodscentscompany.com, accessed on 28th August 2022). [3] Values at/near saturation point appeared as constant numbers.

Table S4. Aroma and palate attributes evaluated during sensory analysis.

Attributes	Definition
fruit aroma	Intensity of the overall fruit aroma
smoke aroma	Perception of any type of smoke aroma, including smoked meat/bacon, toasty, charry, cigar-box, estery
cold ash aroma	Burnt aroma associate with ashes, including ashtray, tarry, campfire
earthy aroma	Any aroma associated with musty, dusty, wet-wood, barnyard, mushroom-like, dank, moldy, stagnant, stale
medicinal aroma	Aromatic characteristic of Band-Aids, disinfectant-like, including cleaning products, solvents, chemicals
burnt rubber aroma	Perception of burnt rubber-like aromas
reduced aroma	Perception of stinky, rubber, sulfur, garlic aromas
oxidized aroma	Perception of vinegar or bruised apple aroma
fruit flavor	Intensity of the overall fruit flavor
smoky flavor	Perception of smoke flavor, including bacon and smoked meat
medicinal flavor	Perception of medicinal flavors, including disinfectant-like, cleaning products and solvents
burnt rubber flavor	Perception of burnt rubber flavor
reduced flavor	Perception of stinky, rubber, sulfur, garlic flavor
oxidized flavor	Perception of vinegar or bruised apple flavor
ashy aftertaste	Length of taste associated with residue of ashtray perceived in the mouth after expectorating, including coal ash, ashtray, tarry, acrid, campfire
woody aftertaste	Length of taste associated with woody residue, includes wood, oak, pencil shavings
metallic	The ‘tinny’ flavor associated with metals
acidity	Intensity of sour/acid taste
bitterness	Intensity of bitter taste or aftertaste
saltiness	Intensity of salty taste
hotness	Intensity of warmth/heat due to ethanol
drying	Intensity of drying sensation in the mouth
astringency	Intensity of rough, puckering mouthfeel

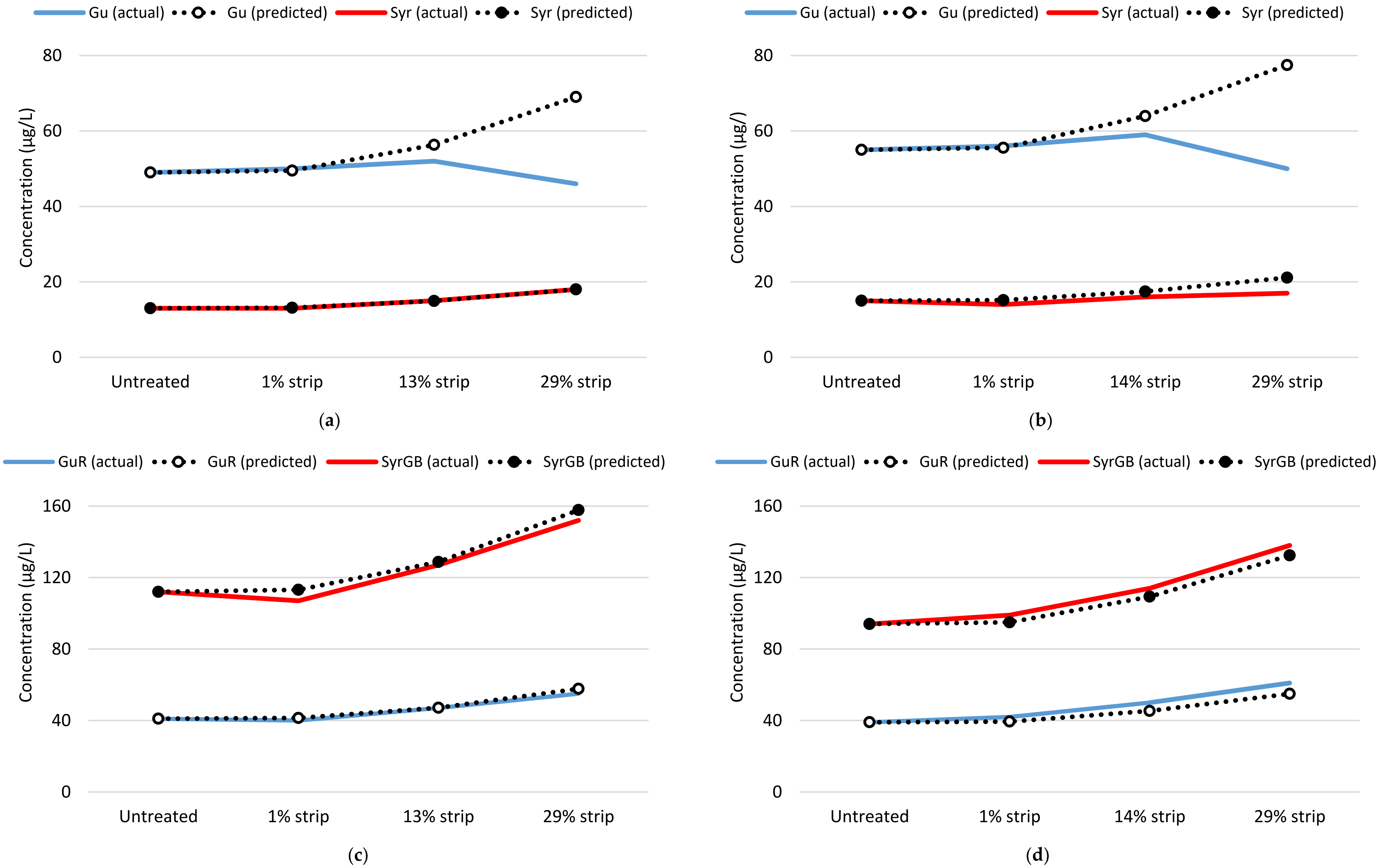

Figure S1. Predicted and actual concentrations of (**a**,**b**) guaiacol (Gu) and syringol (Syr), and (**c**,**d**) their glycoconjugates (GuR and SyrGB) , in smoke-tainted (**a**,**c**) Shiraz Sangiovese and (**c**,**d**) Petit Verdot Sangiovese wines, before and after SCC distillation. Predicted concentrations were calculated by adjusting initial (untreated) concentrations based on 1%, 13–14% and 29% stripping rates (i.e., accounting for concentration due to removal of 1%, 13–14% and 29% of the initial wine volume).
